# Calcium imaging reveals depressive- and manic-phase-specific brain neural activity patterns in a murine model of bipolar disorder: a pilot study

**DOI:** 10.1038/s41398-021-01750-8

**Published:** 2021-12-07

**Authors:** Min Chen, Hongjun Tian, Guoyong Huang, Tao Fang, Xiaodong Lin, Jianmin Shan, Ziyao Cai, Gaungdong Chen, Suling Chen, Ce Chen, Jing Ping, Langlang Cheng, Chunmian Chen, Jingjing Zhu, Feifei Zhao, Deguo Jiang, Chuanxin Liu, Guangchuan Huang, Chongguang Lin, Chuanjun Zhuo

**Affiliations:** 1 Micro-imaging Center of Psychiatric Disorder, Institute of Mental Health, Jining Medical University, 272013 Jining, China; 2 Key Laboratory of Real Time Imaging of Brian Circuits in Psychiatry and Neurology (RTIBNP_Lab), Tianjin Fourth Center Hospital Affiliated to Nankai University, Tianjin Fourth Center Hospital; Tianjin Mental Health Center of Tianjin Medical University, 30022 Tianjin, China; 3 Center of Psychiatric Animal Model, Institute of Mental Health, Wenzhou Seventh Peoples Hospital, 325000 Wenzhou, China; 4 Department of Psychiatry Medical Center, Wenzhou Seventh Peoples Hospital, 325000 Wenzhou, China; 5 Department of Clinical Laboratory, Wenzhou Seventh Peoples Hospital, 325000 Wenzhou, China

**Keywords:** Bipolar disorder, Depression

## Abstract

Brain pathological features during manic/hypomanic and depressive episodes in the same patients with bipolar disorder (BPD) have not been described precisely. The study aimed to investigate depressive and manic-phase-specific brain neural activity patterns of BPD in the same murine model to provide information guiding investigation of the mechanism of phase switching and tailored prevention and treatment for patients with BPD. In vivo two-photon imaging was used to observe brain activity alterations in the depressive and manic phases in the same murine model of BPD. Two-photon imaging showed significantly reduced Ca^2+^ activity in temporal cortex pyramidal neurons in the depression phase in mice exposed to chronic unpredictable mild stress (CUMS), but not in the manic phase in mice exposed to CUMS and ketamine. Total integrated calcium values correlated significantly with immobility times. Brain Ca^2+^ hypoactivity was observed in the depression and manic phases in the same mice exposed to CUMS and ketamine relative to naïve controls. The novel object recognition preference ratio correlated negatively with the immobility time in the depression phase and the total distance traveled in the manic phase. With recognition of its limitations, this study revealed brain neural activity impairment indicating that intrinsic emotional network disturbance is a mechanism of BPD and that brain neural activity is associated with cognitive impairment in the depressive and manic phases of this disorder. These findings are consistent with those from macro-imaging studies of patients with BPD. The observed correlation of brain neural activity with the severity of depressive, but not manic, symptoms need to be investigated further.

## Introduction

Bipolar disorder (BPD) is a chronic psychiatric disorder affecting 1–4% of the global population [1]. With advances in multiple research techniques, the pathological features of BPD have been increasingly examined from different perspectives [2–10]. Based mainly on magnetic resonance imaging (MRI) and electroencephalographic (EEG) findings, the macro-brain connectivity hypothesis holds that brain circuit connectivity disturbance in the emotion and reward networks in bipolar illness can guide larger-scale efforts to understand how the human brain architecture impacts mood regulation in patients with BPD [11–18]. Micro-brain connectivity evidence, provided mainly by molecular studies based on the brain development hypothesis and performed with animal models, suggests that homeostatic structural plasticity is disturbed in BPD [19–31]. Moreover, accumulating studies based on the monoamine theory suggest that the dopamine transporter, oxytocin, and dopamine systems act in tandem to regulate corticostriatal circuitry, and that this synergistic interaction is perturbed in patients with BPD [17–50]. Thus, the current evidence suggests that disturbances of macro- and micro-brain connectivity and monoamine transmitters are the pathological features of BPD, and that these features are caused by neuronal axon, dendrite, brain transmitter, and brain electrical activity dysfunction, as well as genetics and epigenetics (reciprocal gene–environment interactions) [20–55]. More importantly, macro- and micro-brain connectivity is correlated due to the disturbance of neural synapse connections in BPD [32–50].

Few studies, however, have examined manic/hypomanic or depressive phase-specific pathological features of BPD [37, 38]. Moreover, dynamic alterations in brain functional activity, cognition, and behavior during the switching phase of BPD have rarely been examined in the same patient or animal model. Such research can provide important information about the mechanism underlying phase switching, ultimately guiding the targeting of treatment for patients with BPD and prevention high-risk individuals with family histories of the BPD [48–56]. In recent years, in vivo two-photon calcium imaging has been used to investigate brain pathological features in animal models of neocortical processing disorder and neurological and mental diseases [46–54]. In addition, brain alteration patterns have been found to differ among the stages of BPD progression [45–56].

Hence, in the present pilot study, we used in vivo two-photon imaging to observe brain activity alterations during the depressive and manic/hypomanic phases in a murine model of BPD. As clinical studies have shown that depressive episodes usually precede manic episodes in patients with BPD, we established the model with induction of the depressive phase by chronic unpredictable mild stress (CUMS) application [57], followed by manic-phase induction by ketamine injection [58–60]. We hypothesized that: (1) in vivo calcium imaging would demonstrate impaired neural activity in the temporal cortex (TPC) and prefrontal cortex (PFC), (2) this impairment would have phase-specific patterns, (3) these patterns would be associated with differences in cognitive and behavioral performance between BPD phases, and (4) the patterns would be related to macro- and micro-brain connectivity features.

## Materials and methods

### Animals and experimental design

Male C57BL/6 mice aged 4–5 weeks were housed in groups in a standard animal facility with free access to food and water. An adeno-associated viral (AAV) vector expressing GCaMP6s, a fluorescent calcium indicator, was injected stereotaxically into the TPC and PFC of each mouse. We investigated neural transmission in the TPC and PFC because these loci are the seats of higher emotional and cognitive function and have been implicated in the pathogeneses of depression and mania [47–71]. After recovering from this procedure, the mice were divided randomly into two groups: negative controls with no exposure or treatment and BPD mice. The investigators were blinded to the groups during experiments. The ethics committees of Wenzhou Seventh and Tianjin Fourth Center hospitals approved this study (IRB-2020-animal-BPD-001 and IRB-2020-TJFCH-004, respectively), and all procedures were performed in accordance with the hospitals’ ethical standards.

The BPD model was designed to mimic a protocol for mania prevention [55–60]. Following our previous work, we established it by exposing the mice to CUMS using a standard protocol to provoke depressive behavior; 1 day later, we initiated a course of daily intraperitoneal injection of ketamine (25 mg/kg) to provoke manic behavior [68–71]. Assessments were performed after the establishment of each phase.

### Stereotaxic injection

Anesthesia was induced with 1.25% avertin, the scalp was incised and locally sterilized, and the periosteal tissue was removed. A stereotaxic instrument (RWD, China) was used to identify the hindlimb region of the primary somatosensory cortex (S1HL; about 0.5 mm anterior to bregma and 1.5 mm lateral). An injection hole was created on the cranium with a high-speed microdrill (OmniDrill35; WPI, Jerusalem, IL, USA), and a glass microelectrode connected to an ultra-micro-injection pump (Nanoliter 2010; WPI) was used to inject 80 nl AAV2/1-hSyn-GCaMP6s or 150 nl AAV2/1-hSyn-DIO-GCaMP6s (>1 × 10^13^ gene copies/ml; University of Pennsylvania Gene Therapy Program Vector Core) into the fifth cortical layer at a 60° angle to avoid imaging site damage. The glass electrode was kept in the brain tissue for a total of 5 min [72, 73].

### In vivo two-photon calcium imaging

Three weeks after stereotaxic injection, the mice were anesthetized with 1.25% avertin, the skull was exposed, and two metal bars were attached to the rostral and caudal portions of the skull, respectively, with glue (Loctite 401) and dental cement to ensure head restraint during imaging. One day later, a high-speed microdrill was used to create an imaging window above S1HL. A glass coverslip was applied to the window using Vetbond tissue adhesive (3M, USA).

In vivo calcium imaging was performed on awake mice under head restraint using a 920-nm excitation laser with a water-immersed objective (× 20, 1.1 numerical aperture; Zeiss, Germany). Under an LSM780 two-photon microscope (Zeiss), calcium activity was recorded at 2 Hz for 2.5 min at the apical tufts (0–80 μm from pia), vasoactive intestinal polypeptide somas and axons (200–300 μm from pia), somatostatin somas and axons (400–500 μm from pia), and layer 5 pyramidal neuron somas (600–650 μm from pia). These regions of interest were defined manually. Calcium-signal time series were corrected using the TurboReg plugin for ImageJ software (National Institutes of Health, Bethesda, MD, USA). Mean pixel values were averaged to obtain fluorescent (*F*) values, normalized as (∆*F* – *F*_0_)/*F*_0_, where *F*_0_ (the baseline value) was the average obtained during the first 10% of recording. Total integrated calcium values were calculated by summing ∆*F*/*F*_0_ values for the entire time series. Calcium spikes were defined as ≥3 standard deviation increases [74, 75].

### Behavioral assays

The animals were subjected to a sucrose preference test as described previously [76–79], followed by a prepulse inhibition (PPI) test adapted for the quantification of sensory gating function [80, 81]. After acclimation of the mice in a sound-isolating chamber with 65 dB background noise, a 75-dB prepulse (PP) was applied for 20 ms, followed 100 ms later by a 40-ms 120-dB startle stimulus (PA). The mice completed three trials with intervening intervals of 30 s. Scores were averaged and the PPI ratio was calculated as (PA – PP)/PA × 100% [76–81].

### Statistical analysis

Sample size was determined based on previous published data as sufficient to obtain statistical significance. No randomization was performed. Data are presented as means ± standard errors of the mean, unless specified otherwise. Data were compared using one-way analysis of variance and post hoc Tukey tests. Data analysis and figure plotting were performed with the GraphPad Prism software (version 8.0) [65, 66]. No animals were excluded from the present study.

## Results

### CUMS and ketamine exposure evoked abnormal cortical transmission and behavior

**Fig. 1:**
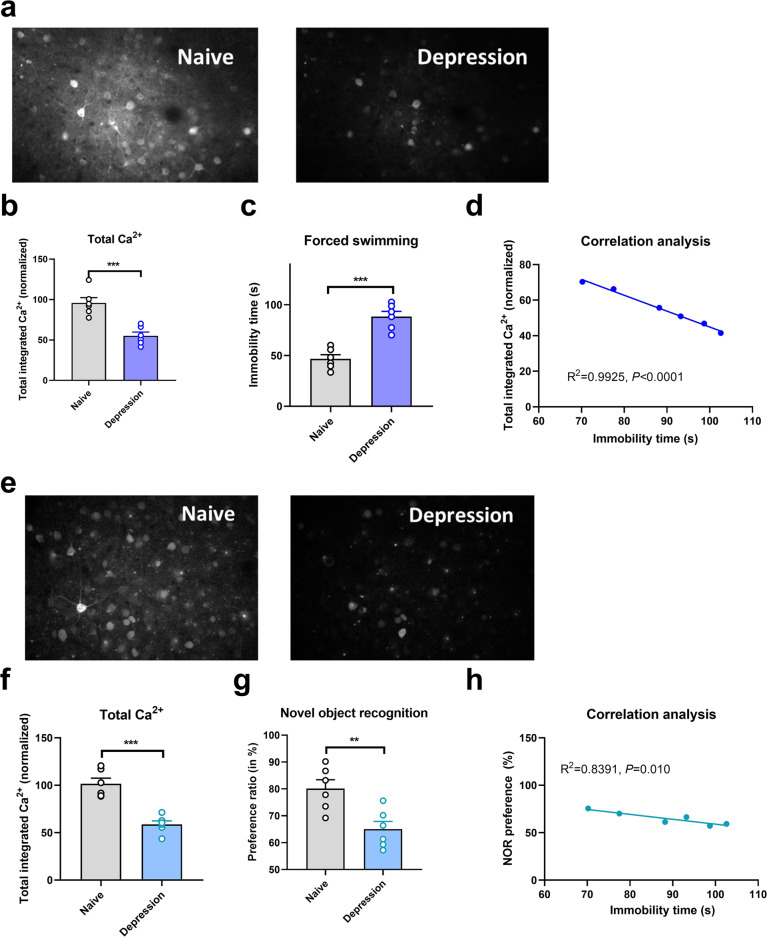
Brain neural activity and the correlation of Brain neural activity and depressive symptoms in the depressive phase of the murine model of bipolar disorder. **a** Brain Ca^2+^ activity in naive murine model; **b** Brain Ca^2+^ activity in the depressive phase in the bipolar disorder murine model; **b"** Normalized total integrated Ca in the depressive phase in the bipolar disorder murine model; **c** Immobility time in the manci phase in the bipolar disorder murine model; **d** The correlation between normalized total integrated Ca and immobility time in the depressive phase in the bipolar disorder murine model. **e** Comparison of the Ca between naïve murine model and the depressive phase of bipolar disorder in the depressive phase of the murine model of Bipolar disorder; **f** Normalized total integrated Ca in the depressive phase in the bipolar disorder murine model; **g** Percentage of preference ratio of the depressive phase in the bipolar disorder murine model; **f** Normalized integrated Ca activity comparison between the naive murine model and depressive phased in the murine model of bipolar disorder; and **h** The correlation between normalized total integrated Ca and immobility time in the depressive phase in the bipolar disorder murine model.

**Fig. 2:**
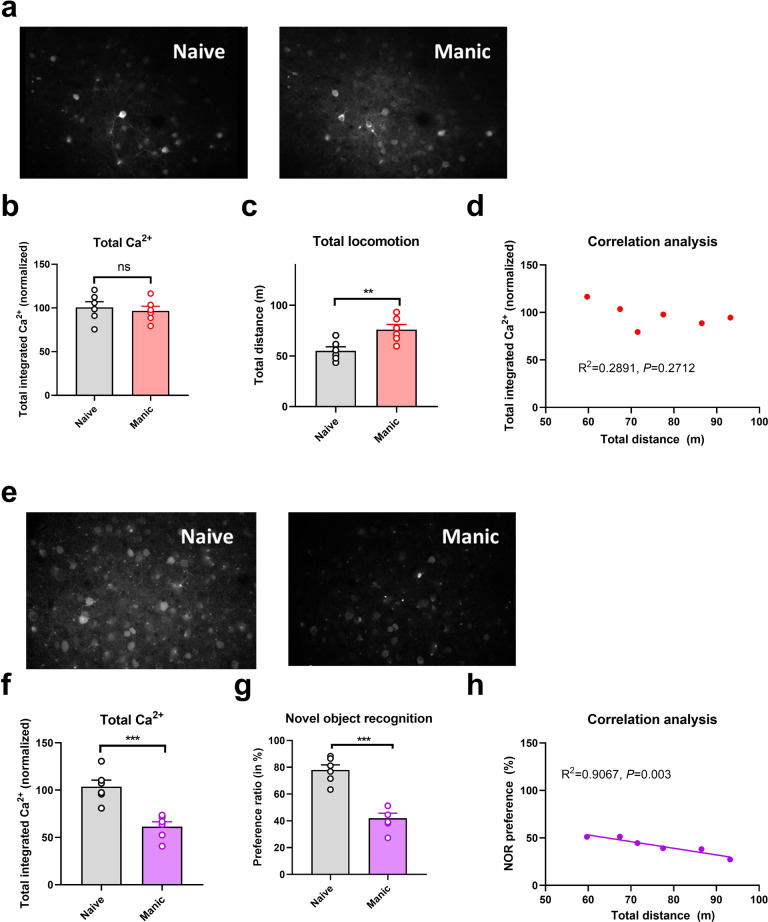
Brain neural activity and the correlation of Brain neural activity and depressive symptoms in the depressive phase of the murine model of bipolar disorder. **a** Brain Ca activity in naive murine model; **b** Bain Ca activity in the manic phase in the bipolar disorder murine model; **b"** Normalized total integrated Ca in the manic phase in the bipolar disorder murine model; **c** Immobility time in the manci phase in the bipolar disorder murine model; **d** The correlation between normalized total integrated Ca and immobility time in the manic phase in the bipolar disorder murine model. **e** Comparison of the Ca between naïve murine model and the manic phase of bipolar disorder in the manic phase of the murine model of Bipolar disorder; **f** Normalized total integrated Ca in the manic phase in the bipolar disorder murine model; **g** Percentage of preference ratio of the manic phase in the bipolar disorder murine model; **f** Normalized integrated Ca activity comparison between the naive murine model and manic phased in the murine model of bipolar disorder; and **h** The correlation between normalized total integrated Ca and immobility time in the manic phase in the bipolar disorder murine model.

Compared with controls, mice exposed to CUMS and ketamine had significantly longer immobility times in the depression phase (*P* < 0.001; Fig. 1c) and significantly greater total distances traveled in the manic phase (*P* < 0.001; Fig. 2c). Two-photon calcium imaging demonstrated significantly less Ca^2+^ activity in TPC pyramidal neurons in the depression phase, but no significant abnormality in the manic phase, in these mice (Figs. 1a, b and 2a, b). Compared with the controls, Ca^2+^ hypoactivity was observed in the PFC in the depression and manic phases in mice exposed to CUMS and ketamine (Figs. 1e, f and 2e, f). Total integrated calcium values correlated significantly with immobility times in the depression phase, but not the manic phase (*P* < 0.001, Fig. 1d; *P* < 0.001, Fig. 2b), due primarily to decreased calcium spike frequencies (*P* < 0.001, Fig. 2c, d). In both phases, exposed mice had smaller novel object recognition preference ratios than did controls (*P* < 0.001; Figs. 1g and 2g). This ratio correlated negatively with the immobility time in the depression phase and with the total distance traveled in the manic phase (Figs. 1h and 2h). Thus, mice exposed to CUMS and ketamine showed dual phenotypes of depression and manic behavior largely consistent with the behavioral expression of patients with BPD, reflecting successful BPD model establishment. They exhibited impaired cognitive ability in the depressive and manic phases, which was more severe in the depressive phase.

### Relationships among brain Ca^2+^ activity, symptom severity, and cognitive impairment

Brain Ca^2+^ activity was related negatively to the severity of depression (Fig. 1d). A similar, but nonsignificant, trend was observed for the severity of mania (Fig. 2d). The relationships between symptoms and cognitive impairment were consistent in the depressive and manic phases. Cognitive impairment was more severe and more strongly correlated with symptom severity in the manic phase than in the depressive phase.

## Discussion

In this pilot microimaging study conducted with a murine model of BPD, we observed significantly reduced brain Ca^2+^ activity in the depressive and manic phases, which is consistent with macro-imaging findings suggesting that the disruption of the emotional network contributes to mood dysregulation in patients with BPD [20–23]. We also observed cognitive impairment in the depressive and manic phases, which was related to the severity of depressive and manic symptoms. In addition, it was more severe and correlated more strongly with symptom severity in the manic phase. Neural activity impairment did not correlate with manic symptom severity in our BPD model.

Numerous reports, especially those on macro-imaging (functional MRI and EEG) studies, describe decreased brain activity in the TPC in the depressive and manic phases of BPD. For example, Cerullo et al. [82]. reported decreased middle temporal gyrus activation during depressive episodes in patients with BPD and those with unipolar depression. Xiao et al. [83]. reported reduced cortical regional homogeneity (ReHo) in the superior temporal gyrus and increased ReHo in the cerebellum in the manic phase in patients with BPD relative to a euthymic group. The ENIGMA Bipolar Disorder Working Group observed widespread bilateral patterns of reduced cortical thickness in the frontal, temporal, and parietal regions among 6503 adults with BPD [32]. These findings, along with our microimaging findings, indicate a reduced ability to adjust the intrinsic emotional network in animal models of BPD and patients with this disorder.

Our observation that brain Ca^2+^ activity was related negatively to the severity of depressive, but not manic, symptoms may be related to the pathological mechanism of BPD. Macro-imaging studies have documented clear correlations of symptom severity with brain functional and structural impairment [84–89]. In addition, macro-imaging techniques may not be able to capture brain features in a timely manner during manic or depressive episodes; in other words, a time lag may affect the results. We could not explore this possibility in this study because we did not use EEG or MRI. A few EEG studies have revealed correlations between brain impairment and symptom severity, and a few animal models of BPD have investigated the relationship between depressive or manic symptom severity and brain impairment. Research involving the simultaneous application of multiple technologies is needed to clarify this relationship.

Clinical and macro-imaging studies have shown that cognitive impairment is a pivotal symptom in patients with BPD; similar findings have been obtained with animal models of depression or mania [90–94]. Our finding that cognitive impairment was more severe in the manic phase provides a new clue for investigation of this difficult-to-improve symptom in patients with BPD, although it may have been influenced by confounding factors. We also found that the mice had more difficulty maintaining attention to complete cognitive tasks during the manic phase in this study. This finding is similar to those from macro-imaging studies of patients with BPD [95–99]. A multiple-arm study is needed to clarify this question.

The lack of a relationship between brain activity and behavioral expression in the manic phase in this study may be related to the regional neurological differences observed in murine models of BPD. For example, Hindley et al. [100] reported that GABAergic system-related genes influence neuronal structure and function in the frontostriatal reward system; we did not examine the striatal region in the present study. Moreover, a recent BPD model revealed the involvement of cerebrospinal fluid proteins in neuronal cell–cell and cell–matrix interactions, particularly in the developing brain, and in pathways of importance for lithium’s mechanism of action [101]. These findings demonstrate the need for more exploration of the central nervous system processes implicated in BPD, and the relationship between the neurological bases of this disorder and behavioral expression [102].

### Limitations

This study has several limitations. First, our findings may be attributable to our induction of the manic phase immediately after the depressive phase in this study, which most closely models the rapid-cycling or mixed-episode form of BPD [61, 62]. An intervening interval of time may have allowed for brain recovery, avoiding a “double hit” effect of the CUMS and ketamine treatments. However, this dual-modeling approach has not been attempted in previous studies, and experience from clinical practice suggests that most patients with BPD switch phases rapidly; some patients even have mixed manic/depressive episodes. Patients with rapid-cycling or mixed-episode BPD show more serious brain activity and cognitive impairments, with no relationship between the two [91, 103–105]; this evidence seems to support our postulation that our model design caused a floor effect, but multiple-arm studies are needed to clarify this issue. In future research of this type, we will seek to identify a better method for BPD modeling to allow the investigation of neural mechanisms underlying other subtypes of this disorder. Second, we used only a few behavioral and cognitive indices, although these indices are classic for animal models. The development of additional tests for the assessment of cognitive alterations might aid more detailed description of the pathological mechanisms of BPD. Third, with this pilot study we could not gain a precise and full understanding of the neural mechanisms underlying BPD, or identify a potential biomarker for the investigation of this disorder in patients. The brain is a highly sophisticated network related to genes and composed of many sub-networks under dynamic development from infancy to advanced age. Our observation of brain Ca^2+^ activity after CUMS may partially explain the lack of a relationship between brain functional impairment and behavioral expression in the subsequently induced manic phase. However, we believe that the brain functional impairment caused by CUMS is a feature of the pathological mechanisms underlying BPD. Finally, unknown factors may have influenced the results of this study, given our limited knowledge of the pathological mechanisms of BPD. Further research on the phase-specific brain pathological features of BPD in animal models and patients is needed.

## Conclusion

In this study, we investigated the pattern of brain Ca^2+^ activity impairment, and the relationships of Ca^2+^ activity and cognitive performance impairment to depressive and manic symptoms and their severity, in a murine model of BPD. The findings are consistent with those of macro-imaging studies of patients with BPD [31–35], implicating intrinsic emotional network disturbance in this disorder and confirming the association of brain neural activity with cognitive impairment during the depressive and manic phases. Brain neural activity correlated with the severity of depressive, but not manic, symptoms in this study. Given the limitations of this pilot study, however, further research is needed to confirm our observations.
